# Similar patterns of linkage disequilibrium and nucleotide diversity in native and introduced populations of the pea aphid, *Acyrthosiphon pisum*

**DOI:** 10.1186/1471-2156-10-22

**Published:** 2009-05-26

**Authors:** Jennifer A Brisson, Sergey V Nuzhdin, David L Stern

**Affiliations:** 1 Section of Ecology and Evolution, University of California at Davis, Davis, CA, USA; 2 Computational and Molecular Biology, University of Southern California, Los Angeles, CA, USA; 3 Department of Ecology and Evolutionary Biology, Princeton University, Princeton, NJ, USA; 4 Current address: Computational and Molecular Biology, University of Southern California, Los Angeles, CA, USA

## Abstract

**Background:**

The pea aphid, *Acyrthosiphon pisum*, is an emerging genomic model system for studies of polyphenisms, bacterial symbioses, host-plant specialization, and the vectoring of plant viruses. Here we provide estimates of nucleotide diversity and linkage disequilibrium (LD) in native (European) and introduced (United States) populations of the pea aphid. Because introductions can cause population bottlenecks, we hypothesized that U.S. populations harbor lower levels of nucleotide diversity and higher levels of LD than native populations.

**Results:**

We sampled four non-coding loci from 24 unique aphid clones from the U. S. (12 from New York and 12 from California) and 24 clones from Europe (12 alfalfa and 12 clover specialists). For each locus, we sequenced approximately 1 kb from two amplicons spaced ~10 kb apart to estimate both short range and longer range LD. We sequenced over 250 kb in total. Nucleotide diversity averaged 0.6% across all loci and all populations. LD decayed slowly within ~1 kb but reached much lower levels over ~10 kb. Contrary to our expectations, neither LD nor nucleotide diversity were significantly different between native and introduced populations.

**Conclusion:**

Both introduced and native populations of pea aphids exhibit low levels of nucleotide diversity and moderate levels of LD. The introduction of pea aphids to North America has not led to a detectable reduction of nucleotide diversity or increase in LD relative to native populations.

## Background

One important goal of evolutionary biology is to identify the genetic causes of phenotypic variation in wild populations. In theory, in large, randomly mating populations where all loci are in linkage equilibrium, genotype can be connected to phenotype by assaying every DNA polymorphism and associating those polymorphisms with phenotypic variation [1]. In practice, loci are often in linkage disequilibrium (LD) and not all polymorphisms can be surveyed due to cost. LD can be exploited, however, to identify genomic regions, rather than individual nucleotides, that are associated with phenotypic variation [e.g., [2]]. The scale at which polymorphisms must be sampled depends on the level and decay of LD across the genome. Higher levels of LD allow shallower sampling of polymorphisms, but provide reduced resolution for locating causal variants. Lower levels of LD force denser sampling of polymorphisms, but provide greater precision for linking genotype to phenotype.

Systematic studies of LD have been performed for few species and mainly for plants. Among plants, LD varies widely, with higher levels observed in predominately selfing organisms such as *Arabidopsis thaliana *[3,4] and soybeans [5]. Selfing reduces effective heterozygosity and therefore the ability of recombination to reduce LD. Among outcrossers such as sunflowers [6] and maize [7,8], LD decays more rapidly, reaching background levels within one kilobase. In humans LD persists from tens to hundreds of kilobases [2,9,10]. In a flycatcher (*Ficedula albicollis)*, significant LD extends over 400 to 500 kb [11]. In *Caenorhabditis elegans *LD generally decays to insignificant levels within a kilobase [12,13] and in *Drosophila melanogaster *within even shorter distances [14].

Here we characterize levels of LD and nucleotide diversity in the pea aphid (*Acyrthosiphon pisum*). Pea aphids, like most aphids, are cyclical parthenogens [15]. During the spring and summer months, females are parthenogenetic, giving live birth to typically genetically identical daughters via a modified meiosis lacking genetic recombination [16] (but see [17] for a recent review of the possibility of genetic variation within an aphid clone). A lineage of asexual females is typically referred to as a clone. Pea aphid males and sexual females are produced asexually in the fall in response to shortened day-length and lower temperatures [18,19]. Eggs produced by the sexual generation diapause through the cold winter months when adults perish.

The pea aphid genome has been sequenced at 6× coverage by the Human Genome Sequencing Center at Baylor College of Medicine and an assembly has been released . Notable aspects of the pea aphid's biology include switching seasonally between asexual and sexual reproduction [15], harboring obligate and facultative bacterial endosymbionts [20,21], producing winged and unwinged morphs in response to environmental cues [22], displaying strong preferences for different host plants [23], transmitting plant viral diseases [24], and producing overwintering, diapausing eggs [25]. Many of these traits exhibit variable phenotypes that can be explored using a mapping approach, which would be informed by estimates of levels of nucleotide diversity and LD in this species.

Pea aphids infest field crops of the pea family such as alfalfa (genus *Medicago*) and clover (genus *Trifolium*). Although Eurasian in origin, the pea aphid has been introduced to North America, presumably via agriculture, probably many times over the last 200 years [26]. Severe bottleneck events, such as those associated with the introduction of only a few individuals, are expected to dramatically decrease levels of nucleotide diversity and increase levels of LD [27,28]. Less severe bottlenecks decrease the number of low-frequency single-nucleotide polymorphisms (SNPs) in the introduced population [29]. We therefore hypothesized that introduced populations harbor lower levels of nucleotide diversity and higher levels of LD than native populations. If true, the two populations would have different utility for the coarse-mapping (introduced) and fine-mapping (native) of phenotypic variation.

Aphid clones display increased survivorship and fecundity on their preferred host plants. In New York, pea aphids specialize on alfalfa (*Medicago sativa*) or red clover (*Trifolium pratense*) [23,30] and this specialization has a genetic basis [31]. Specialization on alfalfa, clover and other species of the pea family has also been observed in France [32], Sweden [33], and the United Kingdom [34]. In California, pea aphids specialize on white clover (*Trifolium repens*), but no alfalfa specialists have been reported. Clones found on alfalfa are more generalized in that they are able to feed on either clover or alfalfa [35]. We sequenced four loci from pea aphid clones from both alfalfa and clover in Europe and alfalfa in the U. S. to test whether host plant specialization or population origin was associated with population differentiation at the sampled loci and with different patterns of LD or nucleotide diversity.

## Methods

### Sampling

Twelve pea aphid clones were collected from alfalfa fields in Yolo and Galt counties of central California by T. Leonardo and S. Nuzhdin [35]. Twelve clones were collected from alfalfa fields in Western and Central France (Domagné and Lusignan) by J. C. Simon. Two clones were collected from clover fields in Germany (coordinates 50°54' W 11°33'N and 50°59' W 11°31'N) and ten from clover fields in France (coordinates 48°06' W 1°47'N, as well as Domagné and Lusignan) by J. C. Simon. Finally, 12 New York clones were collected from alfalfa fields in Tompkins county by M. Caillaud and Otsego county by J. Brisson. From each clone, we extracted DNA from two to six females using the Qiagen (Valencia, CA) DNAeasy kit.

### Loci Studied

We assembled four contiguous segments of 10 to 20 kb of genomic DNA using sequences from the NCBI trace archive. Two of these loci, CAA and 380, are located on the X chromosome while the other two, 870 and 41, are on autosomes. Pea aphids have three autosomes and one large X chromosome that appears to contain about a third of the nuclear genome content [16]. Small sequence subsets of these 10 to 20 kb segments were previously identified for the CAA, 380 and 870 loci [31,36], while 41 was developed in this study.

We wanted to assay LD within short distances (less than 1 kb) and longer distances (over about 10 kb), so we designed two sets of PCR primers for each of the four loci. The resulting amplicons from each set were spaced by about 10 kb. Because we had two amplicons per locus and four loci, in total we utilized eight amplicons (from each of the 48 clones). Specifically, our amplicons (sized between 500 and 1000 bp) were separated by 9249 bp for locus 380, by 11371 bp for locus 870, by 9163 bp for locus 41, and by 10684 bp for locus CAA. After release of the first assembly of the pea aphid genome, Acyr 1.0, we confirmed these distances, with locus 380 on contig 7746 in scaffold 5539, 870 on contig 14350 in scaffold 14350, 41 on contig 6316 in scaffold 12009 and CAA on contig 18522 in scaffold 15751. We also confirmed that all four of the loci are in intergenic regions. Primers are listed in Table 1. 50 μl PCR reactions included 25 μl of 2 × PCR Master Mix (Fermentas, Glen Burnie, MD), 5 μl of each 10 μM primer, 2 μl of genomic DNA, and 13 μl H_2_O. All PCR reactions were done with negative controls under the following conditions: an initial 95°C anneal, 30 cycles of 95°C for 30 seconds, 55°C for 30 seconds and 72°C for 1 minute, with a final 10 minute extension at 72°C. PCR products were purified using the Invitrogen (Carlsbad, CA) Purelink PCR purification kit and sequenced directly from both ends as well as with the internal primers listed in Table 1.

**Table 1:** Primers used for PCR and sequencing.

Locus	Forward primer	Reverse primer
CAA-1	TAAACGTTTCGGTTTCTGTTCC	AGGGATATCCAGGATGGTTTG

CAA-2	TACATTGCGCACTATAAATTAGGG	ATTAAGTCACCAACCTTTTCCAAC

380–1	ACGGTATCGAATCCCCTTAAAAAC	AGATACCGCAGAACTAGTGGAATG

380–2	TAAAGCCGCATCCTATAAACTCTC	TAATACGGTCGTCTGTCACAACTC

380-int1	TTCGAATGTAATTTATTTACCAGG	

380-int2	CCTCTAAATACGGCCCTGATGAACC	

380-int3	AAACTGTTTAACTCACAAAG	

870–1	CTATGTGGATGTTGATACCGAAAG	TTCCGTATGCTATATTTCCGTCTG

870–2	AACTTCTAAATACCGGACAATCTCG	CAAACTGTCCTCTGAGAAATGAGAGG

870-int	TGAAAGCCCAGACGTCGATGCTAGC	

41-1	CTTCTGCCAGTCACAAACAATC	CGATGAAGATCTTTCACTGCAC

41-2	AACTAAGCCGTCTGCGTTACTC	CATTTACACGGTACCGACATTG

### Sequence Analyses

**Figure 1:**
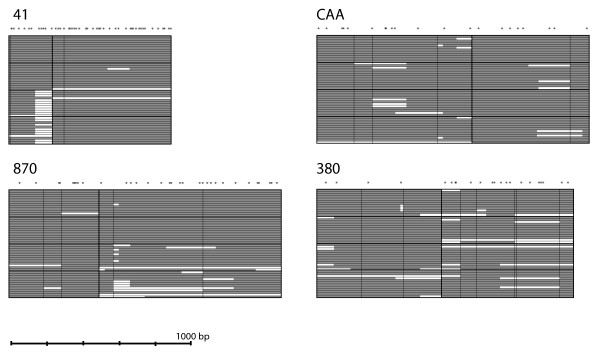
**Summary of the sequence data used in this study for the two autosomal (left, 41 and 870) and two X-linked (right, CAA and 380) loci**. Each locus consisted of two sequenced segments of DNA separated by several kilobases (9.2 kb for locus 41, 11.4 kb for locus 870, 10.7 kb for locus CAA, and 9.2 kb for locus 380). A heavy black vertical line separates the two sequenced segments at each locus. Each locus is additionally broken up into the four different populations, indicated by four horizontal cells separated by heavy black lines. The California population is on top, then the New York, Europe alfalfa, and lastly Europe clover. There are 12 individuals per cell, each separated by a fine black horizontal line. Grey shading indicates that the sequence data were present; white indicates missing data (primarily due to heterozygous indels, see text). Above the boxes are asterisks that specify the approximate sequence position of SNPs. Fine black vertical lines indicate the direct observation of an indel at that position. A scale bar is given at the bottom of the diagram.

Sequences were assembled using CodonCode Aligner (CodonCode Corporation, Dedham, MA). Because these were natural pea aphid isolates, all loci had heterozygous positions and some had heterozygous insertions or deletions (indels). The software identified heterozygous positions that were confirmed by visual inspection of the sequence trace file. In some cases we did not sequence across the entire amplicon due to the presence of heterozygous indels. These areas were treated as unknown data. There were also instances where we were unable to amplify a locus in a given genotype. Figure 1 illustrates the gaps in our data for each of the four loci. All indels were excluded from analyses of nucleotide diversity. Sequences have been deposited in GenBank under accession numbers FJ825706–FJ825751 and FJ858381–FJ858708.

Analyses of LD were performed using the Genetics package [37] in the R statistical computing environment [38]. Our data are genotypic, not haplotypic data. Thus, for two loci, A and B, AB/ab is indistinguishable from Ab/aB. The frequency of AB, p_AB_, in this package is estimated via maximum likelihood, and then used to compute the difference in expected versus observed allele pairs, D, where D = p_AB _- p_A_p_B _and the correlation coefficient between the pairs, r, where r = -D/(√(p_A_p_a_p_B_p_b_)). We thus report LD values between pairs of sites using the squared allele frequency correlation measure, r^2^. Rare sites (where only one individual differed from the others) were excluded prior to computations. Significance of LD between SNPs was assessed using Fisher's exact test, also implemented using the Genetics package. To estimate the decay of LD over distance, we used nonlinear regression of LD between polymorphic sites versus physical distance [7]. The expected value of r^2 ^using a drift-recombination model is E(r^2^) = 1/(1 + C), where C equals the population recombination parameter (C = 4N_e_r). Adding mutation to the model, the expectation becomes:

E(r^2^) = ((10 + C)/(2+C)(11+C))/(1+(3+C)(12+12C+C^2^)/(n(2+C)(11+C)), [39] where *n *is the number of sequences sampled. We fit this model after replacing C by C*distance in base pairs between sites using PROC NLIN in SAS [40].

We calculated π, θ, and Tajima's D using R. π measures nucleotide diversity as the average number of nucleotide differences per site between all pairwise comparisons of sequences and θ is the level of heterozygosity determined by the number of variable positions [41,42]. Tajima's D is a measure of whether the frequency distribution of segregating sites at a locus is consistent with neutral expectations [43]. Levels of population differentiation (F_st_) for each of the four loci were estimated using Genepop [44], which uses a weighted analysis of variance [45]. We also used Genepop to determine whether any of the loci were not in Hardy-Weinberg equilibrium, testing the null hypothesis of random union of gametes using the exact Hardy-Weinberg test [46].

## Results

### Sequence Diversity

To measure levels of sequence diversity and LD decay over short distances (less than 1 kb) as well as over longer distances (about 10 kb), we sequenced two amplicons spaced by about 10 kb at four different genomic regions for 12 clones from New York, 12 clones from California, and 24 clones from Europe (22 clones from France and two clones from Germany). The European clones consisted of 12 alfalfa and 12 clover specialists. Two of the genomic regions were from autosomes (41 and 870) and two were from the X chromosome (380 and CAA). A total of approximately 5452 bp was sequenced for each individual, with over 250 kb sequenced in total. None of the regions included coding sequence and all were in intergenic regions as determined by querying the "nr" (non-redundant) database with a blastX (translated versus protein database) search of their associated genomic contigs. We excluded all indel information from our analyses because we used diploid individuals and could not precisely assay the number or size of indels in some of our individuals. Heterozygous indels in some cases led to missing sequence data (see Figure 1).

Table 2 reports estimates of nucleotide diversity (π) and Watterson's θ. Nucleotide diversity was significantly lower on the X chromosome than the autosomes (1-sided *P *< 0.001, unpaired t-test comparing the eight autosomal π values to the eight X π values; if we increase the sample size contributing to nucleotide diversity estimates by calculating a π value for the North American samples and a π value for the European samples for each locus and then compare the four autosomal π values to the four X π values, 1-sided *P *< 0.01). Further, measures of nucleotide diversity were not significantly different between North American and European populations (NY and CA π values averaged versus Europe, alfalfa and clover averaged: 1-sided *P *= 0.12, paired t-test; again, if we calculate a π value for the North American samples and a π value for the European samples for each locus and compare them, 1-sided *P *= 0.09, paired t-test).

**Table 2:** Measures of nucleotide diversity, Tajima's D, and LD.

Locus	Bp	Population	S^	Pi	Theta	D	Mean r^2 ^short distances	Mean r^2 ^longer distances
41	928	California	26(0)	0.0108	0.0061	2.88**	0.31	0.198

		New York	33(10)	0.0111	0.0087	1.09	0.215	0.187

		Europe, alfalfa	33(17)	0.0091	0.0083	0.37	0.275	0.141

		Europe, clover	38(16)	0.0116	0.0093	0.92	0.193	0.217

870	1487	California	30(13)	0.0061	0.0055	0.45	0.458	0.300

		New York	28(11)	0.0053	0.0049	0.31	0.353	0.044

		Europe, alfalfa	31(2)	0.0093	0.0056	2.54**	0.408	0.167

		Europe, clover	27(4)	0.0072	0.0046	2.19*	0.716	0.159

CAA	1608	California	6(5)	0.0008	0.0010	-0.65	NA	NA

		New York	14(3)	0.0028	0.0020	1.34	0.221	0.182

		Europe, alfalfa	7(3)	0.0024	0.0013	2.45**	0.416	0.447

		Europe, clover	17(2)	0.0040	0.0029	1.28	0.285	0.078

380	1429	California	15(5)	0.0021	0.0019	0.29	0.723	0.039

		New York	14(5)	0.0021	0.0016	1.15	0.565	0.555

		Europe, alfalfa	13(3)	0.0022	0.0016	1.27	0.269	0.076

		Europe, clover	16(3)	0.0031	0.0020	2.00*	0.323	0.22

^Numbers of singleton SNPs indicated in parentheses

* Significant (P < 0.05) and ** highly significant (P < 0.01) Tajima's D values [43]

All loci possessed indels. We observed two 1 bp indels in locus 41. Locus 870 had a 1 bp, a 2 bp, a 3 bp and a 5 bp indel. Locus CAA showed two 1 bp, a 4 bp and a 6 bp indel. Finally, locus 380 had five 1 bp indels, a 4 bp indel, and a 14 bp indel. There were additional indels that we did not directly observe: multiple individuals had sequencing problems at the same region likely due to a heterozygous indel. These can be inferred from Figure 1.

### Tajima's D and Hardy-Weinberg Estimates

Under neutrality, the values of π (based on the number of pairwise differences between individuals) and θ (based on the number of segregating sites) should be equal [42,47]. Tajima's D test compares these two measures of diversity. D values that are significantly different from zero indicate non-neutral evolution resulting from demographic changes or from selection [43]. We calculated Tajima's D for each locus and each population and observed that across all populations and all loci, with only one exception (locus CAA, California), Tajima's D values were positive (Table 2). Five of 16 values were significantly positive at the 95% level. All loci had at least one significant positive D value. Four of the five significant D values were from European populations.

New York and California populations were in Hardy-Weinberg equilibrium for all loci except the autosomal locus 870 (New York: X^2 ^= 94.2, *P *= 0.001, California: X^2 ^= 84.2, *P *= 0.014). Both deviations at locus 870 were towards a deficiency of heterozygotes. All four loci were in Hardy-Weinberg equilibrium within the two European populations.

### Population Structure

F_st _values indicated moderate differentiation between most populations, with an average value of 0.188 (Table 3). The lowest value was observed for the comparison of European alfalfa and European clover populations (0.155) and the highest for the comparison of California and clover-feeding populations from Europe (0.228).

**Table 3:** F_st _values for each pairwise population comparison.

	New York	Europe, Alfalfa	Europe, Clover
Europe, Alfalfa	0.199		

Europe, Clover	0.186	0.155	

California	0.198	0.161	0.228

### Linkage Disequilibrium

**Figure 2:**
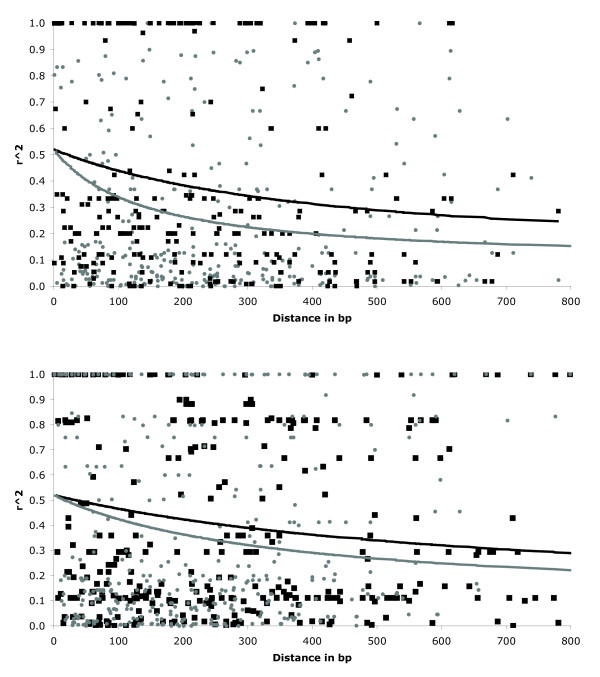
**Plot of the squared correlations (r^2^) versus distance in bp between sites for populations from New York (grey circles) and California (black squares) in the top panel, and Europe, clover (grey circles), and Europe, alfalfa (black squares), in the lower panel**. Data from all four loci (two sequenced amplicons per locus) are included. The fitted curve of Hill and Weir [39] illustrating the expected decay of LD is shown for each population.

**Figure 3:**
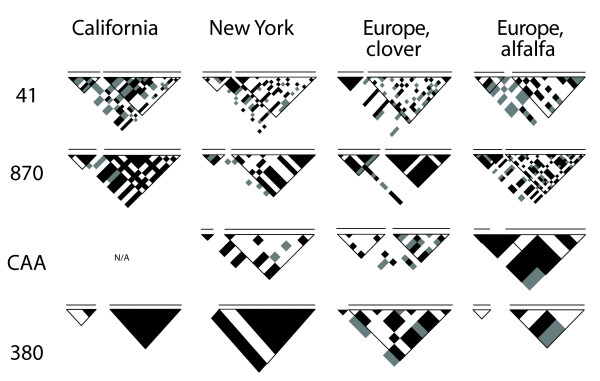
**Significance of pairwise LD between different sites**. Each variable site for a particular locus and population is represented along the left diagonal as well as across the top of each triangle. The significance of the pairwise comparison is represented as a diamond where they intersect, with black *P *< 0.01, grey 0.01 <*P *< 0.05 and white nonsignificant. Each triangle has a different number of diamonds because each locus and population harbored a different number of variable sites. Black lined inverted triangles indicate comparisons within 1 kb (within one of the sequenced amplicons, thus two triangles in most cases), as do the horizontal lines above each graph. Comparisons across approximately 10 kb (between the two sequenced amplicons at each locus) fall outside of those black lined inverted triangles. No pairwise comparisons were computed for the CAA locus for the California population because there was not sufficient nucleotide variation. Refer to Table 2 for total number of variable sites per locus for each of the populations.

We estimated LD levels, as measured by the correlation between variable bases (r^2^), separately for each population and each locus. The average correlation at both short-range (less than 1 kb) and long-range (about 10 kb) distances is listed in Table 2, and the distribution of r^2 ^within a kilobase for all loci pooled is displayed in Figure 2. Figure 3 illustrates the significance of these pairwise comparisons at both distances. Generally, we observed significant levels of LD within 1 kb distances, with an overall average of 0.38, ranging from 0.19 to 0.72. LD decayed slowly within a kilobase (lines in Figure 2), with the fastest rate of decay observed in the New York population. At the larger scale, average r^2 ^values were low relative to the shorter spatial scale with an average of 0.20, although some of these comparisons were still significant (Figure 3).

We also examined levels of LD across loci. Between the two autosomal loci, 870 and 41 (it is unknown whether they are on the same chromosome), the average r^2 ^was 0.15. Loci CAA and 380 on the X chromosome had an interlocus average r^2 ^of 0.18.

## Discussion

### Sequence Diversity and Linkage Disequilibrium

We have investigated the levels of nucleotide diversity and LD across four genomic regions in two introduced pea aphid populations, New York and California, and two host races from native populations in Europe. This is the first estimate of short and longer range LD decay for this species. Previous studies using primarily mtDNA sequence variation, RFLPs, and allozymes have reported low diversity in pea aphid populations [32,48-50]. Like these studies, we find low diversity, with π averaging 0.0056 across all loci and all four populations, ranging from 0.0008 (locus CAA, California) to 0.0116 (locus 41, Europe, clover).

The two loci on the X chromosome exhibit roughly half the diversity of the two loci on the autosomes. These lower X chromosome values may result, at least in part, from the lower effective population size for the X chromosome. In a population with equal numbers of males and females there are three X chromosomes for every four autosomes because females are XX and males are XO. Under neutrality, nucleotide diversity is directly proportional to effective population size [51]. However, these X chromosome π values remained lower than the autosomal values even after multiplying by 4/3 to compensate for the X to autosomal population size differences. If this pattern remains consistent across autosomal and X chromosome loci, then this implies that other factors, such as selection, may act differentially on the X chromosome and autosomes [52].

In a previous study [53], we sequenced portions of the coding regions from 27 different loci from an average of ten individuals from disparate geographical locations. Of these 27 loci, 16 exhibited silent site variation. The average diversity of this silent site polymorphism was 0.0053 (ranging from 0.0006 to 0.0114), very similar to the average of 0.0056 we report here for noncoding variation despite low levels of codon bias in the pea aphid [54].

The levels of noncoding DNA diversity in the pea aphid are two and a half times lower than the diversity estimates of 0.0153 for intergenic regions of *Drosophila simulans *[55] and 0.0142 for noncoding regions of *Aedes aegypti *[56]. Although it is possible that the pea aphid has a lower mutation rate, the more likely explanation for this comparatively low diversity is small effective population size. The pea aphid has a large absolute population size during its parthenogenetic phase in the spring and summer months, but many individuals are genetic clones and thus effective population size is much lower. Biased sex ratios [57], high variance in male mating success [58] and low survival of eggs over the winter might cause additional reductions in effective population size. Finally, as pea aphids are pests on field crops, it is likely that many populations have experienced declines due to pesticides, further reducing the effective population size.

We also examined levels of LD at two scales: within a kilobase, and across approximately 10 kb. Generally, LD is high and decays slowly within a kilobase (Fig. 1). Over ~10 kb, LD has declined to levels only slightly higher than LD averages across loci and therefore what we consider background levels of LD. Thus, this limited survey provides no evidence for long range LD in any of the four populations surveyed. This suggests that LD mapping in the pea aphid will require a relatively high density of molecular markers distributed at the scale of about a kilobase.

### Native Versus Introduced Populations

Pea aphids were introduced to North America, probably from Europe [26]. They are mild pests on field crops such as alfalfa and clover, and have likely been introduced to North America several times over the last 100 to 200 years via agricultural routes. If only a few individuals were introduced at a time, the North American populations would likely exhibit decreased levels of variation relative to native populations [59]. We therefore hypothesized that we would observe lower levels of variation in North American populations relative to European populations. This does not appear to be the case, as both π and θ are similar between the two regions. Likewise, LD levels are similar in populations from both regions. We conclude that native and introduced populations will not have differential utility in LD mapping efforts.

It is unknown how many times or where the pea aphid was introduced into North America. It is possible that repeated introductions have occurred, supplementing the molecular diversity provided by earlier introductions. Consistent with this model, the introduced populations are not depauperate for nucleotide diversity. Further, Tajima's D values are largely positive which can be indicative of admixture, a reduction in population size, or balancing selection [43]. It seems unlikely that balancing selection is acting on all four of these non-coding loci. We therefore favor the hypothesis that the positive Tajima D values reflect demographic factors.

### Host plant specialization

Pea aphids are specialized to a variety of host plants in Europe, including alfalfa and clover [32,33]. Similarly, pea aphids in New York demonstrate strong host plant specialization [23,30], whereas in California both specialists and generalists exist [35]. The specialization in New York either represents introduction of both specialists from the native range, or introduction followed by a secondary event of specialization. All New York individuals were sampled from alfalfa. F_st _values between New York and European alfalfa and between New York and European clover populations are similar (average of 0.199 and 0.186, respectively), providing no evidence for the source of the New York populations. Both of these values are higher than for the comparison of European clover and alfalfa specialists (F_st _= 0.155). This value is only slightly higher than the level of F_st _reported by Simon et al. [32] for comparisons of clover and alfalfa specialists in France either by allozymes (F_st _= 0.098) or microsatellites (F_st _= 0.104).

The California pea aphids examined here were sampled from alfalfa, but all are generalists in that they can feed on both hosts (T. Leonardo, pers. comm). Interestingly, population differentiation is high between California and the European clover clones (F_st _= 0.228), while lower in comparison with the European alfalfa clones (F_st _= 0.168). Although this difference is not statistically significant (one-tailed t-test, *P *= 0.19) the trend of lower differentiation between California and the alfalfa specialists leads us to hypothesize that the host plant generalist aphids found in California are derived from European alfalfa specialists, but that subsequently there has been interbreeding with other introduced populations. Further tests of this hypothesis will require more extensive population sampling and marker development but will provide insight into the interplay of invasion of a new habitat and the gain or loss of host-plant specialization.

## Conclusion

The recent release of the of the pea aphid genome sequence will facilitate mapping efforts aimed at identifying loci underlying variable phenotypes unique to this species, such as polyphenisms and host plant specialization. To inform these future studies, here we provide the first systematic study of nucleotide diversity and LD in both native and introduced populations of the pea aphid. Further, we tested and rejected the hypothesis that bottlenecks associated with the importation of pea aphids into North America have resulted in introduced and native populations being valuable for different mapping needs (coarse versus fine-scale mapping, respectively), finding low levels of nucleotide diversity and moderate levels of LD in all of the populations sampled.

## Authors' contributions

JAB and DLS conceived of the study, JAB carried out the experiments, JAB and SVN performed the analyses, and all three authors participated in the writing of the manuscript. All authors read and approved the final manuscript.
